# Editorial: NAD Metabolism and Signaling in Plants

**DOI:** 10.3389/fpls.2020.00146

**Published:** 2020-02-26

**Authors:** Alisdair Fernie, Shin-nosuke Hashida, Kazuya Yoshimura, Bertrand Gakière, Zhonglin Mou, Pierre Pétriacq

**Affiliations:** ^1^ Department of Molecular Physiology, MPI of Molecular Plant Physiology, Potsdam-Golm, Germany; ^2^ Environmental Science Research Laboratory, Central Research Institute of Electric Power Industry (CRIEPI), Abiko-shi, Japan; ^3^ Department of Food and Nutritional Science, College of Bioscience and Biotechnology Chubu University, Kasugai, Japan; ^4^ Institute of Plant Sciences Paris-Saclay (IPS2), UMR 9213/UMR1403, CNRS, INRAE, Université d’Evry, Université Paris-Diderot, Université Paris-Sud, Sorbonne Paris-Cité, Saclay Plant Sciences, Orsay, France; ^5^ Department of Microbiology and Cell Science, University of Florida, Gainesville, FL, United States; ^6^ Université de Bordeaux, INRAE, UMR BFP, Plateforme Bordeaux Metabolome, Villenave d’Ornon, France

**Keywords:** plant, NAD signaling, pyridine nucleotide, NAD metabolism, fruit, cyanobacteria, light responce

Since its discovery more than a century ago and the conferral of several Nobel laureates for ground-breaking works concerning the molecule, nicotinamide adenine dinucleotide (NAD) is recognized as a fascinating metabolic cornerstone for all living organisms (Berger et al., 2004). This ubiquitous pyridine nucleotide (and its phosphorylated relative, NADP) is an energy cofactor and signal-carrying molecule exerting vital functions in plant metabolic pathways and regulatory processes (Hashida et al., 2009; Gakière et al., 2018b; Gakière et al., 2018a). Molecular studies on NAD involved transcript, protein, and metabolite profiling, and revealed a particularly complex signaling network that can impact not only the core biochemistry of the plant (e.g. photosynthesis, respiration, energy), but also responses to various stress, more importantly immune responses (Zhang and Mou, 2009; Pétriacq et al., 2012; Pétriacq et al., 2016; Alferez et al., 2018; Hashida et al., 2018). Remarkably, compelling evidence indicate that NAD can be released into the extracellular space, where it is sensed by cell surface receptors (Wang et al., 2017; Wang et al., 2019). However, the detailed mechanisms by which NAD acts as a regulator of plant physiology are still poorly understood. This Research Topic gathers some cardinal knowledge on the functions of NAD in plants.

Two papers in the issue focus on the key enzyme NAD kinase which phosphorylates NAD(H) forming NADP(H) (Gakière et al., 2018a)_._ The first review by Ishikawa and Kawai-Yamada examined the roles of the enzyme in cyanobacteria—the evolutionary ancestor of land plant chloroplasts which has a far simpler level of compartmentation, in the oxidative pentose phosphate pathway, the glycolysis, and the pyridine nucleotide transhydrogenase proton translocation system. Survey of 72 cyanobacterial genomes revealed that 69 express a typical NAD kinase, raising the intriguing questions how typical NAD kinases contribute to NAD(P)(H) homeostasis in cyanobacteria and how this is regulated in the species containing the atypical NAD kinases. Also intriguing is the genetic identification of one of the latter as a factor linked to fast growth (Ungerer et al., 2018). However, a number of open questions remain, mainly why cyanobacteria need multiple NAD kinases and what the specific roles of the paralogs are. Further biochemical work alongside that on the membrane permeability of NAD(P)H will go a long way to addressing these questions.

Secondly, Tai et al. reviewed the interplay of NAD kinases with calmodulin-mediated calcium signaling. They described the importance of the balance between NADP(H) and NAD(H) providing evidence suggesting that in response to allosteric regulation NAD kinase is a key regulator of the NADP(H)/NAD(H) ratio (Li et al., 2018). They then listed the evidence that calmodulin activates NAD kinase before performing a detailed analysis of wheat calmodulins, including predictions of gene and protein structures alongside subcellular localization and structure function studies. This provided a comprehensive overview of the putative regulatory control these proteins exhibit on NAD kinase (Tai et al.). Next, the review described the feedback regulation by NAD^+^ derivatives, which is necessary for maintaining the dynamic balance of the cellular metabolites that ensure the normal operation of life activities.

Under sunlight, photosynthetic electron transfer chain is the primary source of NADPH as reducing power for assimilation of CO_2_ by the Calvin cycle. However, under stress conditions that weaken enzyme activity involved in the Calvin cycle, decline of NADPH usage and NADP^+^ recycling occurs and photosynthetic electron flow can be overloaded resulting in generation of reactive oxygen species. Thus, the NADP(H) pool size and redox state are crucial for the chloroplastic redox balance. An important fact is that, at the beginning of photosynthesis, NADP^+^ supply control is dominated by *de novo* NADP^+^ synthesis rather than recycling from the Calvin cycle. In addition, the activity of NADP^+^ synthesis and the NADP pool size varies depending on the light conditions and the ferredoxin-thioredoxin system, by which NADP^+^ is reduced to NADPH. NAD^+^ is exclusively produced in the cytosol, although NADP^+^ is produced at *on demand sites* (cytosol, chloroplasts, and peroxisomes) by various isoforms of NAD kinase. Therefore, the regulatory mechanism of cytosolic NAD^+^ supply is also involved in the chloroplastic NADP^+^ supply control. Hashida and Kawai-Yamada summarized the regulatory mechanisms of NADP^+^ production, focusing on the interactions, crosstalk, and co-regulation between chloroplasts and the cytoplasm at the level of NAD^+^ metabolism and molecular transport.

NAD metabolism underlies a number of NAD-processing reactions. To start recycling NAD from nicotinate (NA), plants developed various strategies to overcome NA toxicity. Enzymatic conjugation of NA (e.g. glycosylation, methylation) has been discovered before and current metabolic analysis with much higher resolution and selectivity become available to examine NAD catabolites using mass spectrometry. Liu et al. identified two novel NA conjugates using a novel method, NA *N*-pentoside and NA *N*-rhamnoside. Interestingly, they provided quantitative profiles of NAD-related metabolites in 24 plant tissues and biochemically characterized the responsible enzymes for conjugation with NA, being a key advance in our understanding of physiological significance of various NA conjugations.

NAD is also important for crops including fruits that constitute an important part of human diets, providing minerals, vitamins, fibers, and antioxidants that are essential for human health. Understanding of the biosynthetic production of these nutrients has attracted considerable attentions in recent years. Although it is well known that fruit development is associated with dramatic metabolic changes (Decros et al., 2019), how NAD metabolism and signaling are regulated during fruit development remains unclear. Decros et al. tackled this question *via* investigation of the changes in NAD(P) pools, transcriptome, and proteome profiles during nine growth stages of tomato fruit. The investigation revealed two critical links during tomato fruit development: a link between fine-tuned NAD(P) homeostasis and profound reprogramming of NAD(P) metabolism and a link between NAD(P)-dependent enzymes and central metabolic pathways, particularly those involved in mitochondrial functions (Decros et al.). These results set up the stage for more in-depth investigations of the implication of NAD(P) in fruit development.

Hence, this Research Topic benefits the scientific community by providing new insights into the many aspects of NAD metabolism and signaling. Several open questions remain in order to understand fully the regulatory circuitry of NAD metabolism and signaling.

## Author Contributions

All authors wrote and/or reviewed the editorial.

## Conflict of Interest

The authors declare that the research was conducted in the absence of any commercial or financial relationships that could be construed as a potential conflict of interest.
